# A Case of Hyponatremia-induced Seizures in an Infant Secondary to Water Intoxication from the Use of Almond Milk

**DOI:** 10.7759/cureus.5899

**Published:** 2019-10-13

**Authors:** Jessica Houck, Latha Ganti, Ariel E Vera

**Affiliations:** 1 Emergency Medicine, University of Central Florida College of Medicine / Hospital Corporation of America Graduate Medical Education (HCA GME) Consortium, Kissimmee, USA; 2 Emergency Medicine, Envision Physician Services, Orlando, USA

**Keywords:** pediatric seizures, hyponatremia, water intoxication, status epilepticus

## Abstract

Pediatric seizures are a common presentation to the emergency department. It is important to separate non-febrile seizures from febrile seizures, as non-febrile seizures have a much broader differential diagnosis. For infants less than six months of age with a normal exam, hyponatremia is the leading cause of new onset non-febrile seizure. Most commonly, this is secondary to water intoxication from inappropriate feeding practices. This case report will review the initial workup of new onset non-febrile seizures in an infant and treatment recommendations for seizures secondary to hyponatremia.

## Introduction

Pediatric seizures are common - approximately 5% of children will have a seizure by the age of 16. The most common cause of pediatric seizure is a benign febrile seizure. Therefore, pediatric patients presenting with seizure should be categorized as febrile versus non-febrile. Non-febrile seizures have a wider differential diagnosis and require a much broader workup. The history and physical exam is a very important aspect of this workup [1].

The differential should start very broad in pediatric patients with new-onset non-febrile seizures. Differential diagnosis will include metabolic derangements including hypoglycemia, non-accidental trauma, intracranial lesions, sepsis, and toxic ingestions. Physical exam should look for signs of trauma, microcephaly, skin findings suggestive of genetic syndromes such as ash leaf spots (tuberous sclerosis) or cafe au lait spots (neurofibromatosis), or hepatosplenomegaly which would be suggestive of metabolic derangements such as glycogen storage diseases [2]. Initial workup of new onset seizure in an infant less than six months of age may include point-of-care glucose, complete blood count, comprehensive metabolic panel, cat scan of the head, urine drug screen, and urinalysis.

In a retrospective chart review of infants less than six months of age that presented to an urban pediatric emergency department with seizure, hyponatremia was found to be the underlying etiology in 70% of afebrile infants with an otherwise normal physical exam [3]. The study had a population size of 47. Seizures from hyponatremia were more likely to be associated with status epilepticus, emergency intubation and hypothermia [3]. Having a temperature of 36.5 degrees Celsius or less was found to be statistically significant for its association with hyponatremic seizures versus all other causes of seizure. Most commonly, hyponatremia in infants is secondary to water intoxication from inappropriate feeding practices by parents [4]. Infants in particular are at risk for water intoxication due to their immature renal function and strong hunger drive.

This case presents an infant with non-febrile seizures secondary to hyponatremia from water intoxication.

## Case presentation

A five-month-old healthy full-term female with a history of sickle cell trait and gastro-esophageal reflux disease presented to the emergency department (ED) with her mother for one episode of nonbloody, nonbilious emesis and somnolence for the past one day. Review of systems otherwise negative. Mother denied fevers, cough, rhinorrhea or trauma. En route, emergency medical services reported that the patient developed seizure with right-sided hypertonicity and left-sided flaccidity and right-sided gaze deviation which was persistent on time of arrival to the emergency department. Intravenous (IV) access was obtained, the patient was placed on a non-rebreather and one dose of IV lorazepam was given with cessation of seizure activity. The patient had a temperature of 35.8°C. She had a normal heart rate and 100% oxygen saturation on room air. Point of care glucose was within normal limits. There was no evidence of trauma, hepatosplenomegaly or abnormal skin findings. Partial sepsis workup initiated with computed tomography (CT) scan of head. A 20 cc/kg normal saline bolus was administered.

While in the ED, the patient had a second seizure which was treated with IV lorazepam successfully. Labs revealed metabolic acidosis with pH 7.28, hyponatremia at 121 mmol/L and a mild lactic acidosis. There was no leukocytosis. Liver function tests were within normal limits. There was no evidence of urinary tract infection. The CT scan of the head was unremarkable and the urine drug screen came back negative.

The patient was given a second bolus of 20 cc/kg normal saline. Further discussion with the mother revealed that the patient had been having issues with reflux and had multiple formula changes without improvement. Approximately three weeks prior to presentation, the mother had discontinued all formula and was giving the patient 9-ounce bottles of approximately half almond milk and half water every 4-5 hours, with an additional 9-ounce bottle of water in between. The mother reported that she did this in an attempt to help with the patient’s reflux symptoms.

On admission, the patient was somnolent but arousable without focal neurologic deficits. She was placed on dextrose 5% ½ normal saline at maintenance rate by the intensivist. A nutrition consult was placed for education to the mother about appropriate feeding practices for an infant. Repeat sodium level came back at 128 mmol/L. The next morning, the patient was awake, tolerating feeds, without neurological deficit. Sodium levels rose to normal limits and stabilized. Maintenance fluids were discontinued and the patient was transferred to the floor and discharged home one day later. There were no further seizure episodes and no evidence of cerebral edema or neurologic sequelae.

## Discussion

Hyponatremia leading to seizures is an increasingly common cause of non-febrile seizures in infants. It is recommended by the Centers for Disease Control (CDC) that cases be reported to the local health department [4]. However, due to lack of reporting, the actual incidence for hyponatremia-induced seizures is unknown.

**Figure 1:**
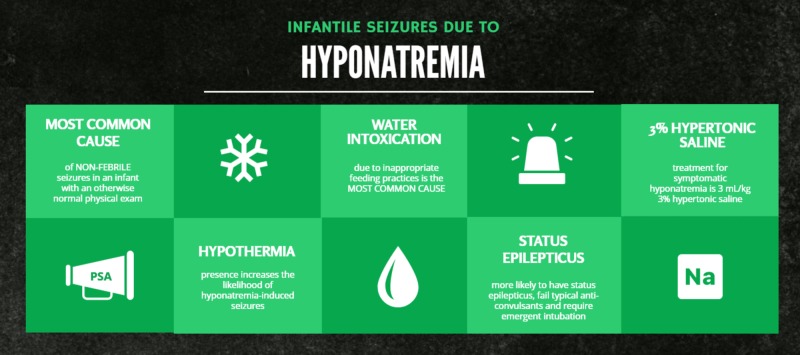
Infantile seizures due to hyponatremia

Signs and symptoms of acute symptomatic hyponatremia include altered mental status (somnolence or irritability), edema, hypothermia and seizures. In a patient with hyponatremia and new onset seizure, treatment should include a 3 mL/kg bolus of 3% hypertonic saline (Figure 1) [5].

These patients are less likely to respond to typical anticonvulsants and more likely to have status epilepticus and require emergent intubation [2]. In the cases that have been reported, prognosis has ranged from complete recovery to permanent debilitation. The more serious outcomes are most likely secondary to prolonged seizures, hypoxia, and direct effects on the central nervous system from the sodium imbalance. The Centers for Disease Control and Prevention also reports multiple cases of hyponatremic seizures in infants secondary to water intoxication [4]. In the cases reported, one of the parents reported giving only water for financial reasons. In another case, the parents reported giving bottles of water in between feeds to help with upper respiratory symptoms [6]. In both cases, the patients had a full recovery.

There has been a push in the health food industry for plant-based milk products, which are low in protein, calories, and fat and high in water content. Most commonly, soya, rice, almond and sweet chestnut milk are used as an alternative to dairy products. There have been many cases of parents substituting these plant-based milk products for formula in the first year of life for various reasons [7-9]. Parents may do this for financial reasons or even to treat a presumed milk allergy and the marketing behind these products has mislead parents to believe that these plant-based beverages are safe alternatives. The consequences of giving infants plant-based milk products can be severe and sometimes devastating. There has also been controversy on whether or not companies should be able to call a product "milk" that are not derived from lactating animals as it can be confusing. One study published in the Archives of Pediatrics Journal in 2014 looked at infants that presented to the hospital between 2008 and 2011 with complications from having plant-based milk diets [8]. These complications included edema secondary to severe protein-calorie malnutrition with hypoalbuminemia, refractory status epilepticus secondary to hyponatremia, failure to thrive with growth arrest, rickets disease from vitamin D deficiency and iron deficiency anemia. One case report published in the Pediatrics journal in 2016 highlighted a case of scurvy in an 11-month-old child who was fed only almond "milk" and almond-based flour products from the age of 2.5 to 11 months [7]. There will likely be more cases of these complications in the future as the popularity for these dairy-alternative beverages increase.

Some suggest that there should be statutory measures forbidding their use in young infants to combat this growing problem. At the very least, there should be campaigns to spread awareness and more education should be provided to parents on the risks of these plant-based milk products. The healthiest, safest option for babies is of course breast milk. However, for those parents that choose not to breastfeed their babies, continued education is needed with regards to appropriate formula choices and dilution.

## Conclusions

It is important to keep a broad differential in infants presenting to the emergency department with non-febrile seizures and this should include metabolic derangements including hypoglycemia, non-accidental trauma, intracranial lesions, sepsis, and toxic ingestions. On physical exam, look for signs of trauma, microcephaly, skin findings suggestive of genetic syndromes such as ash leaf spots (tuberous sclerosis) or cafe au lait spots (neurofibromatosis), or hepatosplenomegaly which would be suggestive of metabolic derangements such as glycogen storage diseases. In non-febrile infants with a normal physical examination, the most common cause of seizure is hyponatremia secondary to water intoxication. The treatment for this is 3 mL/kg of 3% hypertonic saline as well as counselling parents on proper feeding practices for infants.
